# Examining the associations among intraocular pressure, hepatic steatosis, and anthropometric parameters

**DOI:** 10.1097/MD.0000000000017598

**Published:** 2019-10-25

**Authors:** Ying-Jen Chen, Jiann-Torng Chen, Ming-Cheng Tai, Chang-Min Liang, Yuan-Yuei Chen, Tung-Wei Kao, Wen-Hui Fang, Wei-Liang Chen

**Affiliations:** a Department of Ophthalmology, Tri-Service General Hospital; b Department of Internal Medicine, Tri-Service General Hospital Songshan Branch; c Division of Family Medicine; d Division of Geriatric Medicine, Department of Family and Community Medicine, Tri-Service General Hospital, and School of Medicine, National Defense Medical Center, Taipei, Taiwan, Republic of China.

**Keywords:** anthropometric parameters, hepatic steatosis, intraocular pressure

## Abstract

Emerging evidences had reported the positive relationship between obesity and intraocular pressure (IOP). The aim of the present study was to investigate the association between hepatic steatosis and IOP in an adult Taiwanese population.

Seven thousand seven hundred twelve males and 6325 females who received a health examination at the Tri-Service General Hospital during the period from 2010 to 2016 were included in this study.

IOP was measured by noncontact tonometry. Hepatic steatosis was diagnosed by abdominal ultrasound examination. Multivariate regression analyses were used to assess the associations among various anthropometric parameters and IOP.

After adjusting for pertinent covariables, hepatic steatosis had a closer association with increased IOP than percentage body fat, body mass index, or waist circumference (*β* = 0.017, 95% confidence interval [CI] = 0.006, 0.028). This relationship remained significant among males in the study population (*β* = 0.015, 95% CI = 0.001, 0.029). Furthermore, hepatic steatosis was significantly correlated with increased risk of high IOP (odd ratios = 1.235, 95% CI = 1.041–1.465).

Our study highlights that hepatic steatosis is a better index for assessing the relationship with increased IOP than other anthropometric parameters. Underlying pathophysiological mechanisms regulating the association between hepatic steatosis and increasing IOP and even the risk of glaucoma should be examined in further studies.

## 1 Introduction

Development of primary open-angle glaucoma has been reported to be caused by increased intraocular pressure (IOP).^[1–3]^ Generally, the balance between aqueous humor secretion and outflow determines the dynamic change in IOP.^[4]^ Accumulating evidence has shown that increased IOP might be associated with cardiometabolic risk factors, such as type II diabetes mellitus (DM),^[5,6]^ hypertension,^[7,8]^ and other cardiovascular diseases.^[9,10]^ Mori et al demonstrated that obesity was an independent risk factor for increased IOP.^[11]^ Body mass index (BMI) was suggested to have a positive relationship with IOP in previous studies.^[12,13]^ In a large longitudinal study, increased adiposity was significantly associated with elevated IOP in an adult Korean population.^[14]^

Obesity is associated with a spectrum of liver abnormalities, known as nonalcoholic fatty liver disease (NAFLD), characterized by an increase in intrahepatic triglyceride content, known as hepatic steatosis.^[15]^ There is mounting evidence that NAFLD not only complicates obesity but also perpetuates its metabolic consequences. Insulin resistance has been identified as the key aspect in the pathophysiology of NAFLD and metabolic syndrome (MetS).^[16]^ Associations of MetS and its components with high IOP have been reported in previous studies.^[17–19]^ The objective of the present study was to investigate the associations between hepatic steatosis and IOP in a cross-sectional study of an adult Taiwanese population.

## 2 Materials and methods

### 2.1 Study population

During the period from 2010 to 2016, eligible participants were included health examinations at the Tri-Service General Hospital (TSGH). The study design was approved by the institutional review board of TSGH and met the requirements of the Helsinki Declaration. The requirement for informed consent from participants was waived by the institutional review board of TSGH because the data were analyzed anonymously. Exclusion criteria of the study included participants with missing information such as biochemical data, body composition measurement, ophthalmological examination, and abdominal ultrasound examination. There were 14037 eligible subjects included in further analyses.

### 2.2 Ophthalmological examination

TOPCON CT-80 NCT (Abdulrehman Al-Gosaibi GTB, Riyadh, Saudi Arabia) was used to measure IOP in health examinations. All ophthalmological procedures were conducted by well-trained ophthalmologists at the TSGH. Participants who had abnormal fundus findings were excluded at baseline. The IOP measurement was consistently performed in the morning between 8 and 10 am to eliminate the potential interference of diurnal variation.

### 2.3 Diagnosis of hepatic steatosis

Abdominal ultrasound is a useful and reproductive method for evaluating hepatic steatosis.^[20]^ Several diagnostic criteria for hepatic steatosis were used, such as liver to kidney contrast, parenchymal brightness, bright vessel walls, deep beam attenuation, and gallbladder wall definition.^[21,22]^ The diagnosis of hepatic steatosis was established by radiologists based on at least 1 of abovementioned criteria.

We divided participants into 3 groups based on the presence of hepatic steatosis and liver function. Participant who had increased alanine aminotransferase or aspartate aminotransferase (>40 mg/dL) was defined as liver function impairment. Mild grade of hepatic steatosis was determined as the presence of fatty liver based on the ultrasound image without liver function impairment. Moderate to Severe grade of hepatic steatosis was determined as the presence of fatty liver based on the ultrasound image accompanied with impaired liver function.

### 2.4 Measurement of anthropometric parameters

BMI is calculated by a general formula, with the weight in kilograms divided by the square of the height in meters (kg/m^2^). Body fat percentage (PBF) is measured by bioelectric impedance analysis (BIA) (InBody720; Biospace, Inc, Cerritos, CA), which is a commonly used method for assessing body composition because of its ease of use and portability of the equipment.^[23]^ Waist circumference (WC) is measured at the mid-level between the iliac crest and the lower border of the 12th rib.

### 2.5 Covariates

Cigarette smoking in participants was assessed by asking the question “Do you now smoke cigarettes?.” Alcoholic consumption was determined by a self-report questionnaire. Exercise status was defined as having exercise at least 1 time in a week. History of DM and MetS was also obtained from a self-report questionnaire. Systolic blood pressure (SBP) was estimated using a sphygmomanometer when the participants were seated. Biochemical data such as triglyceride (TG), high-density lipoprotein cholesterol (HDL-C), fasting plasma glucose (FPG), and C-reactive protein (CRP) were measured using standard procedures.

### 2.6 Statistical analysis

The relationship between various anthropometric parameters and IOP was analyzed using a linear regression. The association between various anthropometric parameters and risk of high IOP was determined using a logistic regression model. We adjusted these regressions for multivariable models as follows: Model 1 was unadjusted. Model 2 included Model 1, age, gender, TG, HDL-C, SBP, FPG, and CRP. Model 3 included Model 2, exercise status, history of cigarette smoking, alcoholic consumption, DM, and MetS. Statistical significance was defined as a *P*-value of ≤.05. Analyses in the present study were conducted using Statistical Package for the Social Sciences, version 22.0 (SPSS Inc, Chicago, IL) for Windows.

## 3 Results

### 3.1 Demographic characteristics

The eligible participants comprised 7712 males and 6325 females (Table 1); the mean age was 46.88 ± 13.00 and 47.00 ± 12.61 years, respectively. Male subjects had higher BMI and WC and PBF than female subjects. Baseline characteristics such as IOP, SBP, TG, HDL-C, FPG, CRP, exercise status, history of cigarette smoking, alcoholic consumption, DM, and MetS showed significant differences across these 2 groups.

**Table 1:**
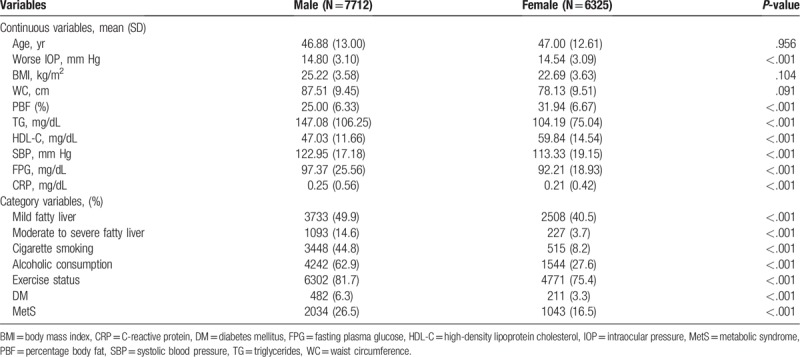
Characteristics of study population.

### 3.2 Associations between various anthropometric parameters and IOP

After adjusting for pertinent covariables, associations between PBF, BMI, WC, and hepatic steatosis and IOP are shown in Table 2. PBF, BMI, and hepatic steatosis had a significant association with IOP in the fully adjusted model, with *β* values of 0.002 (95% confidence interval [CI] = 0.001, 0.003), 0.005 (95% CI = 0.003, 0.006), and 0.018 (95% CI = 0.006, 0.029), respectively. Hepatic steatosis was more closely associated with increased IOP than other anthropometric parameters. However, no significant difference was observed in the relationship between WC and IOP. Furthermore, patients with moderate to severe grade of hepatic steatosis had closer association with increased IOP than mild grade with *β* values of 0.029 (95% CI = 0.010, 0.049).

**Table 2:**
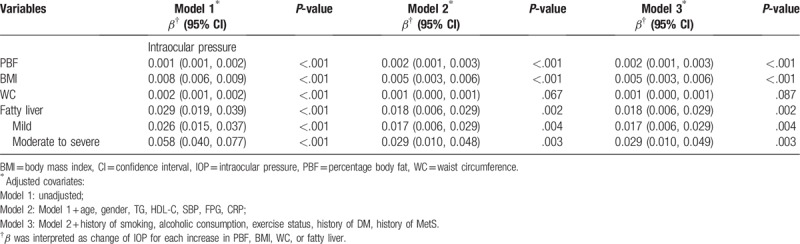
Association between anthropometric parameters and IOP.

In Table 3, we categorized participants into 2 groups by gender. PBF, BMI, WC, hepatic steatosis, and moderate to severe grade of hepatic steatosis were positively associated with IOP in the male study population with *β* values of 0.003 (95% CI = 0.002, 0.004), 0.006 (95% CI = 0.004, 0.009), 0.001 (95% CI = 0.000, 0.002), 0.016 (95% CI = 0.001, 0.031), and 0.030 (95% CI = 0.007, 0.052), respectively. By contrast, no anthropometric parameter had significant associations with IOP in the female study population.

**Table 3:**
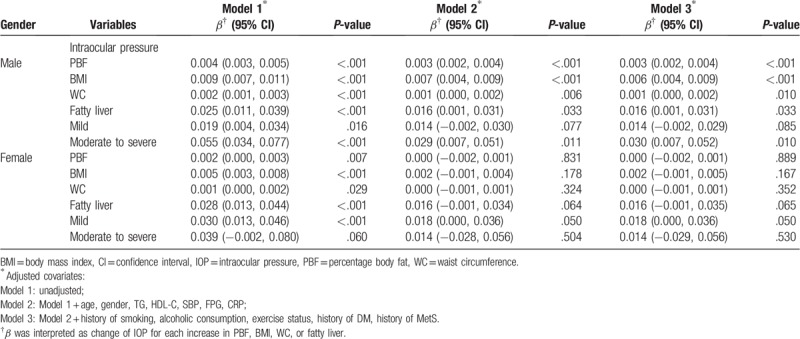
Association between anthropometric parameters and IOP in gender difference.

### 3.3 Associations between anthropometric parameters and risk of high IOP

A multivariate logistic regression model was used to analyze the relationship between various anthropometric parameters and risk of high IOP (IOP >18 mm Hg) (Table 4). Consistent with above results, the significant difference was only observed in the male population. After fully adjusting for covariables, increased PBF, BMI, and hepatic steatosis had significant associations with increased risk of high IOP, with odds ratio (OR) of 1.028 (95% CI = 1.009–1.047), 1.059 (95% CI = 1.027–1.093), and 1.292 (95% CI = 1.008–1.657), respectively. Increased severity of hepatic steatosis had a higher risk for high IOP than other anthropometric parameters. In Table 5, we analyzed the association between 2 grades of hepatic steatosis and the risk of high IOP. Moderate to severe grade had higher risk for high IOP than mild grade with OR of 1.333 (95% CI = 1.000–1.788).

**Table 4:**
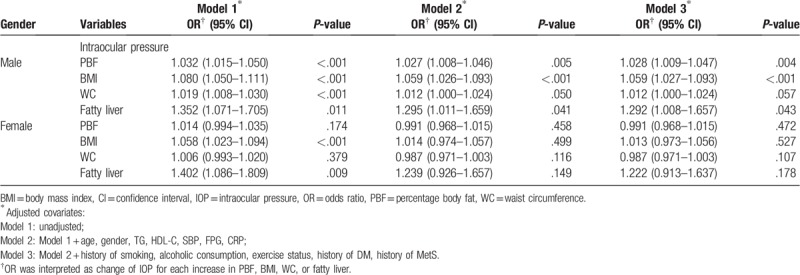
Association between anthropometric parameters and risk of high IOP.

**Table 5:**
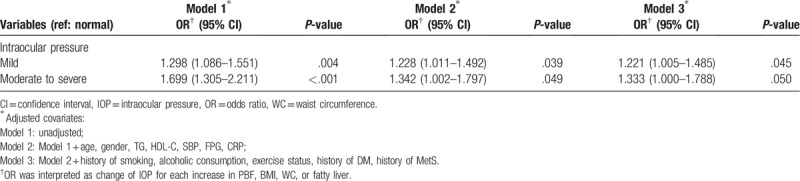
Association between different grades of fatty liver and risk of high IOP.

## 4 Discussion

In the present study, we found that hepatic steatosis was more closely associated with increased IOP than other anthropometric parameters. This relationship remained significant in the male study population but not in females. To date, this research is the first to examine the association between hepatic steatosis and IOP in a general Taiwanese population.

Associations between obesity and IOP have been reported in previous studies. In a large cohort study of Korean adults, adiposity was significantly associated with increased IOP.^[14]^ Mori et al demonstrated that obesity is an independent risk factor for increased IOP in both cross-sectional and longitudinal analyses.^[11]^ A positive relationship was found between BMI and IOP in both genders in a population-based study.^[24]^ In a recent study, a healthy metabolic profile did not protect obese adults from hepatic steatosis and fibrosis, indicating that obesity itself might contribute to liver fibrosis.^[25]^ Our findings demonstrated that hepatic steatosis had a stronger relationship with IOP than other obesity indices, suggesting that alterations in glucose, fatty acid and lipoprotein metabolism might play an important role in determining IOP levels.

Associations between high serum glucose levels and an increased risk of high IOP have been proposed in prior studies.^[26,27]^ One of the mechanisms proposed is the shifting of excessive fluid into the anterior chamber, caused by a hyperglycemia-induced osmotic gradient.^[17]^ Another proposed etiology of increased IOP is that the trabecular meshwork might be damaged by hyperglycemia.^[28]^ In some studies, corticosteroids have been incriminated in the exacerbation or production of the glaucomatous state. At the light of the role of the endocrine system in the pathogenesis of nonalcoholic fatty liver disease.^[29]^ Higher serum TG is known to increase IOP through the accumulation of orbital adipose tissue, which causes increased orbital and episcleral pressure, thereby decreasing aqueous humor outflow.^[30,31]^ In addition, lower HDL-C was reported to elevate episcleral pressure because of vascular sclerosing changes and increased serum osmolality.^[32]^ Increased oxidative stress, caused by adiposity,^[33]^ was involved with impaired function of the trabecular meshwork and the intracellular system, leading to increased IOP.^[34,35]^

There were several potential limitations among the present study. First, a casual inference between anthropometric parameters and IOP was unavailable due to the cross-sectional design of this study. A longitudinal survey had been suggested for further studies. Second, Goldmann applanation tonometry (GAT), the standard IOP measurement, was not used in our study. Instead, we measured IOP using a noncontact tonometer due to several advantages over GAT, including convenience and noninvasiveness, which enhanced patient cooperation.^[36]^ Third, all IOP tests were performed at a single time rather than over repeated measurements, which failed to represent longitudinal change. Next, analyses of potential confounders such as corneal thickness were not included. Last, diagnosis of hepatic steatosis was made through ultrasonography rather than liver biopsy. There were small differences of sensitivity and specificity between ultrasonography and biopsy.^[37]^ However, the results may be affected because ultrasonography still assesses dead space. In addition, it was not possible to evaluate the fat accumulation in the liver by ultrasound if the percentage is less than 30%.

## 5 Conclusion

In the present study, we found that increased severity of hepatic steatosis was more closely associated with increased IOP than other anthropometric parameters in an adult population attending health examinations in Taiwan. A gender difference was noted in that this relationship remained significant in male subjects. It is important for further research to examine the pathophysiologic associations between hepatic steatosis and IOP. Furthermore, screening for NAFLD and its metabolic components is necessary in patients with increased IOP to improve upon or minimize glaucoma complications.

## Author contributions

**Conceptualization:** Wei-Liang Chen.

**Data curation:** Ying-Jen Chen, Jiann-Torng Chen, Ming-Cheng Tai, Chang-Min Liang, Yuan-Yuei Chen, Tung-Wei Kao, Wen-Hui Fang, Wei-Liang Chen.

**Formal analysis:** Ying-Jen Chen, Jiann-Torng Chen, Ming-Cheng Tai, Chang-Min Liang, Yuan-Yuei Chen, Tung-Wei Kao, Wen-Hui Fang, Wei-Liang Chen.

**Investigation:** Ying-Jen Chen, Jiann-Torng Chen, Ming-Cheng Tai, Chang-Min Liang, Yuan-Yuei Chen, Tung-Wei Kao, Wen-Hui Fang, Wei-Liang Chen.

**Methodology:** Ying-Jen Chen, Jiann-Torng Chen, Ming-Cheng Tai, Chang-Min Liang, Yuan-Yuei Chen, Tung-Wei Kao, Wen-Hui Fang, Wei-Liang Chen.

**Supervision:** Wei-Liang Chen.

**Validation:** Ying-Jen Chen, Wei-Liang Chen.

**Visualization:** Ying-Jen Chen, Wei-Liang Chen.

**Writing – original draft:** Ying-Jen Chen.

**Writing – review and editing:** Ying-Jen Chen, Wei-Liang Chen.
